# Mrs. Elizabeth A. Beard

**Published:** 1883-03

**Authors:** 


The Death of Mrs. Beard.—Mrs. Elizabeth A. Beard, wife of the late Dr. George M. Beard, died of pneumonia at the Grand Hotel, New York, January 31. The week previous her husband died of the same disease.

A Prior Claimant to Galvani's Discovery.—Professor S. Thompson points out the little-known fact that Swammerdam anticipated the famous initial experiment of Galvani by more than a hundred years. Being on a visit in Tuscany, in 1678, the illustrious Dutch naturalist showed to the Grand Duke that when a portion of muscle of a frog's leg, hanging by a thread of nerve bound with silver wire, was held over a copper support so that both nerve and wire touched the copper, the muscle immediately contracted.

